# Aromatic Profile and Phenolic Composition of White Wines from Hybrid Grapes Grown onto Different Rootstocks and Regions of Brazil

**DOI:** 10.1021/acsomega.5c08882

**Published:** 2026-03-03

**Authors:** Guilherme Francio Niederauer, Leila Gimenes, Júlio César Rodrigues Lopes Silva, Marcos dos Santos Lima, Giuliano Elias Pereira, Juliana Rocha de Souza, José Luiz Hernandes, Mara Fernandes Moura Furlan, Roselaine Facanali, Marcia Ortiz Mayo Marques

**Affiliations:** † Instituto Agronômico, Centro de Pesquisa e Desenvolvimento de Recursos Genéticos Vegetais, Av. Barão de Itapura, 1481, Campinas, São Paulo CEP 13020-902, Brazil; ‡ Universidade Estadual de Campinas, Instituto de Biologia, Rua Monteiro Lobato, 255, Cidade Universitária, Campinas, São Paulo CEP 13083-862, Brazil; § Universidade Estadual Paulista, Faculdade de Ciências Agronômicas, Avenida Universitária, 3780, Alto do Paraíso, Botucatu, São Paulo CEP 18610-034, Brazil; ∥ Departamento de Tecnologia em Alimentos,Instituto Federal do Sertão Pernambucano, Campus Petrolina, Rod. BR 407 Km 08, S/N, Jardim São Paulo, Petrolina, Pernambuco CEP 56314-520, Brazil; ⊥ Empresa Brasileira de Pesquisa Agropecuária - Embrapa Uva e Vinho, R. Livramento, 515 - C.P 130 - Centro, Bento Gonçalves, Rio Grande do Sul CEP 95701-008, Brazil; # Instituto Agronômico, Centro APTA de Frutas. Av. Luiz Pereira dos Santos, 1500, Corrupira, Jundiaí, São Paulo CEP 13214-820, Brazil

## Abstract

This study investigated the phenolic and aromatic composition of wines produced from four white grape cultivars grafted onto two rootstocks grown in two distinct regions of São Paulo, Brazil: Votuporanga and Jundiaí. Wines made from Moscato Embrapa (ME) and Moscatel de Jundiaí (MJ) cultivars exhibited the highest total phenolic contents, averaging 278.91 and 238.94 mg/L, respectively. Environmental factors influenced phenolic accumulation, with higher concentrations generally observed in wines from Votuporanga. In contrast, IAC Madalena and IAC Ribas cultivars showed greater stability across sites and rootstocks, with minimal variation in phenolic content. Aromatic composition analysis revealed esters as the dominant volatile class, particularly ethyl octanoate, ethyl decanoate, and ethyl hexanoate, which contribute to the wine’s fruity and floral characteristics. Some volatiles, such as terpinolene and isoamyl acetate, showed cultivar-specific occurrence, affecting sensory characteristics. Principal component analysis (PCA) of volatile organic compounds demonstrated that genetic factors had a stronger influence than environmental or rootstock variables, with apparent clustering by cultivar. High-performance liquid chromatography with diode array detection (HPLC-DAD) identified phenolic acids as the most abundant compounds, with Moscato Embrapa wines showing the highest concentrations, especially caftaric acid. *cis*-resveratrol, a bioactive stilbene, was notably present only in IAC Ribas and IAC Madalena wines. PCA of phenolic compounds further supported the predominance of genetic influence on wine composition, with limited impact from location or rootstock. Overall, the results emphasize that the phenolic and aromatic profiles of wines produced from white grapes are primarily determined by the genetic makeup of the cultivars, with environmental conditions and rootstock playing secondary roles. These findings have implications for grape selection and vineyard management aimed at producing wines with desired chemical and sensory characteristics.

## Graphical abstract

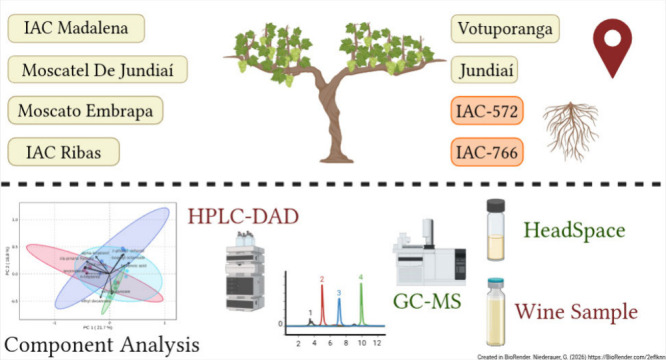

## 1 Introduction

The acceptance of wine on the market is closely related to its organoleptic properties, including aroma, taste, and color. These characteristics result from a wide range of chemical structures of different compounds present in grapes, which influence the potential flavor and aroma of the fruit, directly reflecting on the final product. Among the wine’s aromatic composition, various compounds can contribute to the aroma to different extents.^1^ This contribution is defined based on the odor perception threshold, which indicates the lowest concentration at which a compound can be detected by smell.^2^ Wines with intense and fruity aromas, for instance, typically indicate the presence of numerous esters in their volatile compound content, as these substances are known to contribute to fruity aromas.^3^

On the other hand, anthocyanins and further phenolic compounds contribute to the taste and color of wines.^4^ However, the presence of these compounds in wines represents more than their contribution to the sensory attributes of the drink. The attractiveness of these products and their increasing global consumption are closely tied to the benefits they provide for human health. Several studies point out the therapeutic properties of the phenolic composition in wines, including the antioxidant,^5,6^ antiinflammatory,^7,8^ antimicrobial,^9^ neuroprotective,^10^ and cardioprotective actions.^11^

To obtain the maximum desirable characteristics in a wine, these products must be produced using high-quality grapes. In this respect, several factors influence the grape and wine quality and production, among others, the specific grape variety used in its elaboration (once each variety has an individual chemical composition), the use of appropriate agronomic techniques, and cultural practices adopted in the vineyard, the use of rootstocks that are adapted to the growing conditions and the conditions that the vines are cultivated, such as soil, climate conditions of the region and water availability.^12,13^ Several studies have demonstrated that rootstocks not only influence the characteristics of grapevines but can also have a significant positive impact on the content of phenolic compounds and the antioxidant activity of various grape species.^14–16^ However, this influence depends on the affinity between the canopy and rootstock.^17^

Additionally, climate change has a significant impact on vine development, as this crop is highly susceptible to even slight changes in climatic conditions. Higher ultraviolet (UV) radiation, common in southern hemisphere regions, can influence the biosynthesis of heterogeneous classes of phenolic compounds, such as flavanols, and volatile organic compounds, thereby increasing the accumulation of these compounds in grape berries.^18^ However, when the temperature rises, besides its impact on fruit maturation, there is an increase in sugar content, a decrease in organic acids and total acidity, and an improvement in potassium content. Additionally, climate change can promote the proliferation of certain viticultural pathogens and induce abiotic stress in plants.^19^

From this perspective, in recent years, research institutes such as the Agronomic Institute (IAC) and Brazilian Agricultural Research Corporation (Embrapa) have developed new hybrids and rootstocks for winemaking through their genetic breeding program. These studies aim to develop cultivars more adapted to tropical regions and to enhance resistance to the primary pests and diseases affecting grapevines.^20,21^ Furthermore, research in genetic breeding programs can support future cultivar crossings to achieve the quality attributes of the fruit based on its chemical composition.

Nowadays, Brazilian wines are primarily produced from American grapes, especially those of the *Vitis labrusca* species and its hybrids (*V. labrusca* × *V. vinifera*),^22^ with the majority of the fruit cultivation concentrated in the southern region of the country—this subtropical and temperate region presents average annual temperatures of 17 °C. However, investments in productive techniques, such as grafting, and the development of new hybrid varieties have facilitated the expansion of this cultivar to the tropical regions of the country, including the northwest of São Paulo state, which has a mean annual temperature of 24.1 °C.^23^ The main grapes for wine cultivated in São Paulo are Seibel-2, Isabel, Bordô, Niagara Branca, Niagara Rosada, IAC 138-22 ‘Máximo’, and Moscatel. There was also a slight increase in the cultivation of grapes from *Vitis vinifera*, such as Cabernet Sauvignon, Merlot, and Syrah, and a higher increase of hybrid cultivars, such as Máximo and Moscatel, which represent 18.5% and 11.4% of the number of new plants, respectively.^22^

Thus, the objective of the present study was the evaluation of the aroma profile and the phenolic chemical composition of wines made with the varieties Moscato Embrapa (ME), Moscatel de Jundiaí (MJ), IAC Madalena (M), and IAC Ribas (R), grafted onto the rootstocks ‘IAC 766 Campinas’ and ‘IAC 572 Jales’, grown in two different cities of São Paulo state, Brazil, Votuporanga and Jundiaí.

## 2 Materials and Methods

### 2.1 Chemicals and Reagents

Methanol was supplied by J. T. Baker (Phillipsburg, NJ, USA). Ultrapure water obtained from a Milli-Q system (Millipore, Bedford, MA, USA) was used to prepare all solutions. Standards including gallic, syringic, chlorogenic, ρ-coumaric, caffeic, and *trans*-caftaric acids, (+)-catechin, (−)-epicatechin, (−)-epicatechin gallate, (−)-epigallocatechin gallate, procyanidin B1 and B2, were purchased from Sigma-Aldrich (St. Louis, MO, USA). *Cis*-resveratrol and *trans*-resveratrol were purchased from Cayman Chemical (Michigan, EUA). Quercetin 3-glucoside, rutin, kaempferol, and procyanidin A2 were purchased from Extrasynthese (Genay, France). P.A. Sodium Chloride was supplied by Synth (Diadema, SP, Brazil).

### 2.2 Grape Varieties, Growing Conditions, and Experimental Location

The experiments were conducted at two different cities, located in Votuporanga (20°20′ S and 49°58′ W, latitude 525 m) and Jundiaí (23°17′’ S and 46°9′’ W, latitude 700 to 900 m), São Paulo state, Brazil. According to Köppen’s climate classification,^24^ the climate of Votuporanga is classified as type Aw, indicating a tropical climate with a dry winter. The region of Jundiaí is classified as Cfb, indicating a temperate climate with mild summers. The treatments consisted of a combination of four scion cultivars and two rootstocks, namely Moscatel de Jundiaí, IAC Madalena, Moscato Embrapa, and IAC Ribas, and the rootstocks IAC 572 Jales and IAC 766 Campinas, totaling eight canopy and rootstock combinations (Table 1).

**Table 1:** Codes of Wines Resulted from the Combination of Different Grapevine Varieties, Rootstocks, and Cultivation Sites in São Paulo State, Brazil

	Grape cultivation site/rootstock			
	Votuporanga		Jundiaí	
grape variety (code)	IAC 572	IAC 766	IAC 572	IAC 766
IAC Madalena (M)	M5V	M7V	M5J	M7J
Moscato Embrapa (ME)	ME5V	ME7V	ME5J	ME7J
Moscatel de Jundiaí (MJ)	MJ5V	MJ7V	MJ5J	MJ7J
IAC Ribas (R)	R5V	R7V	R5J	R7J

Moscato Embrapa cultivar was obtained from a cross between ‘Couderc 13’ and ‘July Muscat’ at Embrapa Uva e Vinho in 1983. It is characterized by its high resistance to bunch rot and high fertility, ensuring abundant harvests of fully ripe grapes with sugar content around 19° Brix. The curls are large, conical, and loose; the medium berries have a slight muscatel flavor. The exceptional characteristics of ‘Moscato Embrapa’ enable the production of a semidry white wine, typically aromatic, with low acidity and a pleasant taste that appeals to the Brazilian consumer. The Moscato Embrapa cultivar produces wines with a muscatel flavor, generally characterized as aromatic.^25^

Moscatel of the Jundiaí cultivar was obtained by Inglez de Souza at the Agronomic Institute in 1957, resulting from a cross between Seyve Villard 5276 and Pirovano 4. It has vigorous, productive plants but is vulnerable to mildew, presenting large, long, winged clusters.^26^

IAC Madalena cultivar was developed through a cross between Seibel 11342 and Moscatel de Canelli in 1950. The cultivar exhibits medium to late cycles, and its grapes yield white, aromatic Muscat wines with good acidity, making them suitable for producing sparkling wines.^26^

IAC Ribas cultivar is a hybrid cultivar developed at the São Roque Experimental Station from the Agronomic Institute (IAC) by the researcher Wilson Corrêa Ribas. It is a white grape cultivar characterized by small, round berries and seeds, a neutral flavor, a medium maturation cycle, high productivity, and tolerance to major fungal diseases.^27^ In 2021, this cultivar was registered with the Ministry of Agriculture, Livestock and Supply, Brazil, as ‘IAC Ribas’.^20^

Cultivar IAC 572 Jales, obtained from the cross between *V. caribaea* × 101-14 Mgt by Santos Neto at the Agronomic Institute, was recommended for cultivation in 1970. It is vigorous and grows well in both clay and sandy soils. Its leaves are resistant to primary diseases, and cuttings exhibit excellent rooting and grafting capabilities.^28^

IAC 766 Campinas cultivar was obtained by crossing the 106-8 Mgt × *V. caribaea* rootstock by Santos Neto at the Agronomic Institute. Vigorous, it adapts perfectly to the environmental conditions of São Paulo and northern Paraná, regions where it is widely used. Its leaves are disease-resistant, and its branches overwinter better than those of the IAC 313 Tropical rootstock; its cuttings have excellent rooting rates.^28^

### 2.3 Winemaking

A total of 16 wines were produced through winemaking using various combinations of grape cultivars, rootstocks, and regions in São Paulo state. Table 1 lists the respective codes for each sample. Microvinification of hybrids was performed according to the methodology proposed by Blouin & Peynaud.^29^

### 2.4 Headspace Volatile Analysis Solid-Phase Microextraction-Gas Chromatography (HS-SPME-GC-MS)

HS-SPME was used to analyze the volatile compounds in the 16 wines using a 30 μm Divinylbenzene-Carboxen-Polydimethylsiloxane (DVB/CAR/PDMS) fiber (Stableflex, 2 cm, Supelco, Bellefonte, PA, USA). The fiber was conditioned before each analysis at a temperature of 230 °C for 30 min, and the volatiles were absorbed under the following optimized conditions: 10 mL of each wine was added in a 35 mL headspace vial with a screw cap and Teflon septum, followed by the addition of 3 g of NaCl. The solution was kept under agitation at a constant temperature (30 °C) for 30 min. The volatile compounds were extracted after exposing the fiber to the headspace for 30 min. Then, each sample was analyzed using a QP-5000 gas chromatography coupled with a mass spectrometer (Shimadzu, Kyoto, Japan). Chromatographic separation was performed using a DB-5 column (30 m × 0.25 mm i.d., 0.25 μm film thickness), and the chromatographic conditions were oven temperature program, 35 °C increased at 3 °C/min to 240 °C (total run time of 65 min); split ratio of 1/20. The ion source and MS transfer line were kept at 220 and 230 °C, respectively, and electron ionization was performed at 70 eV. The mass range was set from 40 to 450 *m*/*z* units. Helium (99.9999% of purity) was used as carrier gas at a constant flow rate of 1 mL/min. The analyses were performed in triplicate. CLASS 5000 software (Shimadzu, Kyoto, Japan) was used for instrument control and data acquisition. Compounds were tentatively identified by comparing the substance mass spectra with the NIST 14 database (National Institute of Standards, Gaithersburg, MD, USA) and the linear retention index (LRI) with the literature.^30–32^ GC retention index of each compound was calculated based on the injection of a homologous series of C_8_–C_20_ *n*-alkanes (Merck-St. Louis, MO, USA) using the Van den Dool and Kratz equation.^33^

### 2.5 Total Phenolic Content

The total phenolic content of the wine samples was determined by spectrophotometry using the Folin-Ciocalteu methodology, as described in the literature^34^ and adapted by Arnous et al. (2001).^35^ The measured wavelengths were expressed to mg/L of gallic acid using a calibration curve.

### 2.6 Identification and Quantification of Phenolic Compounds by HPLC-DAD

Analyses were performed using high-performance liquid chromatography (HPLC), an Agilent 1260 Infinity LC (Agilent Technologies, Santa Clara, CA, USA) equipped with a Diode Array Detector (DAD) (model G1315D). Chromatographic separation for phenolic compounds was performed in a Zorbax Eclipse Plus RP-C18 analytical column (100 × 4.6 mm i.d., 3.5 μm) coupled to a Zorbax C18 guard column (12.6 × 4.6 mm i.d., 5 μm). The column temperature was set at 35 °C. Data acquisitions were performed using OpenLAB CDS ChemStation EditionTM software (Agilent Technologies, Santa Clara, CA, USA). The method described by Padilha et al. was used to characterize phenolic compounds. Wines were filtered through 0.45 μm polypropylene filters (Chromafil Xtra, Macherey-Nagel, Düren, Germany) and injected (20 μL). The mobile phases were composed of water acidified with 0.1 M phosphoric acid (pH = 2.0, eluent A) and acidified methanol with 0.5% phosphoric acid (eluent B); a flow rate of 0.8 mL·min^–1^ was used. Elution was complete in 33 min using the following gradient: 0–5 min, 5% B; 5–14 min, 23% B; 14–30 min, 50% B; 30–33 min, 80% B (return to the initial conditions).

### 2.7 Statistical Analysis

The Scott-Knott test was applied to compare total phenolic content among different cities and rootstocks using R.^37^ The aroma and phenolic composition results were subjected to multivariate statistical analysis. Data were autoscaled and subjected to principal component analysis (PCA) and a heatmap. The heatmap was generated based on the Euclidean distance using Ward’s method. Analyses were performed in MetaboAnalyst 6.0.^36^ To test the significance of genetic and environmental factors on chemical composition of the aromatic and phenolic compounds, Permutational Multivariate Analysis of Variance (PERMANOVA) was performed using the adonis2 function from the vegan package in R.^37^ The analysis used Euclidean distance with 999 permutations and tested the effects of variety, rootstock, and location on volatile and phenolic compound profiles. Statistical significance was set at *p* < 0.05.

## 3 Results and Discussion

### 3.1 Total Phenolic Content of Wines

The total phenolic content of wines produced from each combination of scion cultivar, rootstock, and location are shown in Figure 1. The total phenolic content of the wines showed a statistically significant difference, with values ranging from 361.82 mg/mL (ME5J) to 143.41 mg/L (M7J), with an overall average of 216.64 mg/L. The Samples from Moscato Embrapa (ME, blue) and Moscatel de Jundiaí (MJ, purple) showed the highest concentration values, with overall averages of 278.91 and 238.94 mg/L, respectively. Variations in the environment tend to affect specialized metabolism in both qualitative and quantitative terms, leading to increased production of phenolic compounds (Teixeira et al., 2013). The results indicated that MJ and ME cultivars showed a higher difference in the total phenolic content between the cities where the grapes were grown. Values found for wines made with grapes from Votuporanga were higher than those obtained in Jundiaí, except for IAC Madalena­(M) and IAC Ribas­(R) grafted onto the IAC 572 rootstock, which presented a higher value when cultivated in Jundiaí. Despite belonging to the same state in Brazil (São Paulo), the two cities are located 465.8 km apart and have different weather conditions throughout the year, with higher temperatures in Jundiaí and varying precipitation volumes.

**Figure 1:**
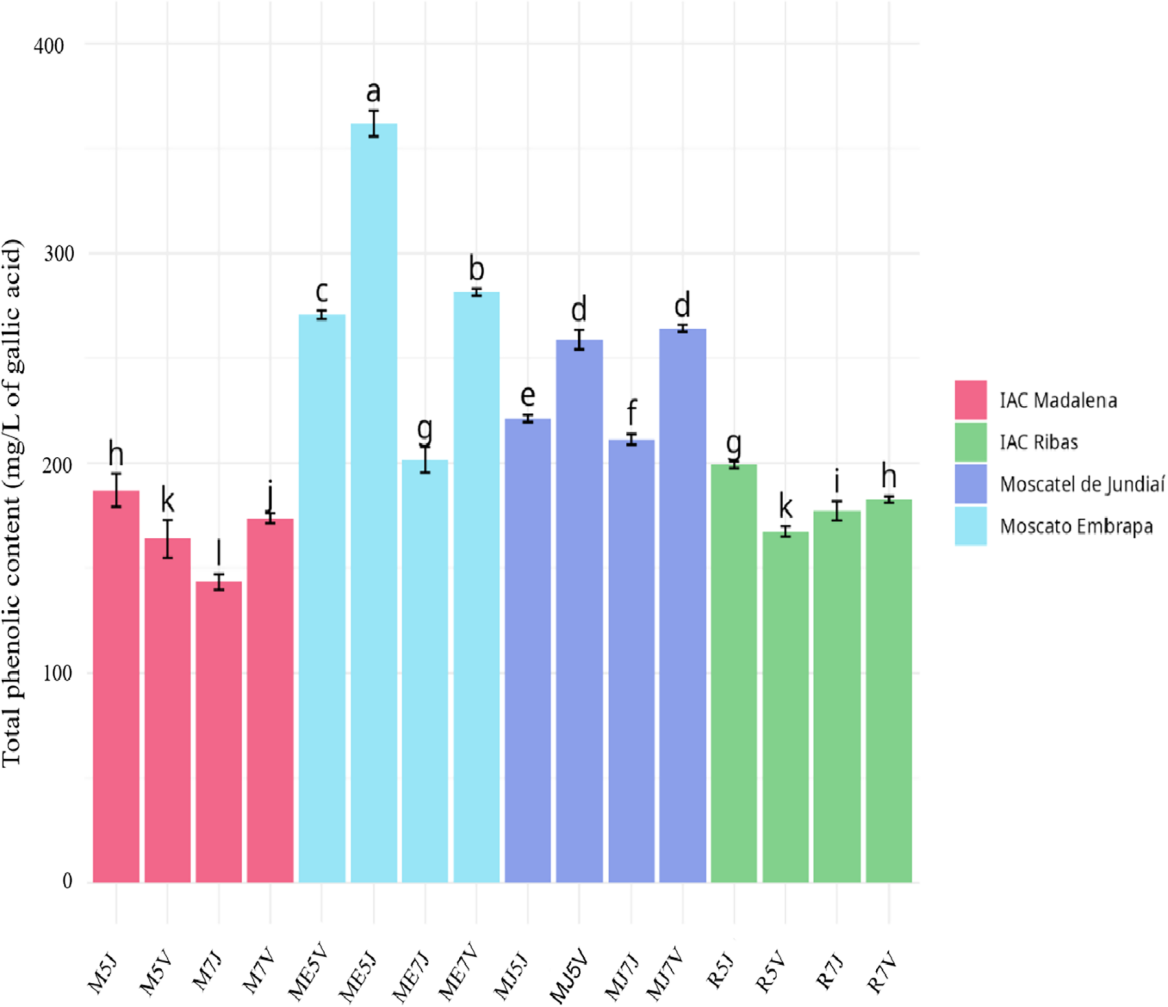
Concentration of total phenolic content in mg/L for each sample as determined by the Folin–Ciocalteu method.

### 3.2 Chemical Composition of Wine Aroma

Table 2 shows the aromatic chemical composition of the 16 analyzed wines. Esters are the major volatiles found in the samples, and ethyl hexanoate, ethyl decanoate, and ethyl octanoate were identified as the most abundant compounds.

**Table 2:** Aromatic Composition of White Wines Made from the Combination of Different Vine Varieties, Rootstocks, and Cultivation Sites, in São Paulo State, Brazil

	LRI^a^		relative content (%)															
compounds	Lit.^b^	Exp.^c^	MJ5V	MJ7V	MJ5J	MJ7J	ME5V	ME7V	ME5J	ME7J	M5V	M7V	M5J	M7J	R5V	R7V	R5J	R7J
**Esters**			93.7	92.9	85.6	93.1	90.0	93.9	94.2	88.7	90.6	98.1	87.9	91.3	90.0	91.9	90.0	69.2
ethyl butanoate	802	801	1.3	1.0	0.7	0.6	1.9	1.1	2.2	1.2	1.4	1.6	1.2	1.0	0.4	0.9	0.4	0.7
ethyl lactate	815	812	0.2	0.1	0.1	-	0.3	0.3	0.4	-	-	0.4	1.1	-	0.3	0.4	0.2	0.3
ethyl isovalerate	849	851	0.2	0.2	0.1	0.2	0.2	0.3	0.1	0.1	-	0.1	0.6	-	0.2	0.2	-	0.2
isoamyl acetate	869	878	8.9	12.3	2.0	-	7.4	6.3	1.9	4.1	0.8	4.0	3.2	2.0	2.2	4.5	3.3	2.0
2-methyl butyl acetate	875	970	0.6	0.9	0.2	0.1	0.2	0.2	0.4	0.1	-	0.1	-	0.7	0.2	0.2	0.2	0.1
ethyl hexanoate	997	1007	14.9	14.4	15.9	24.1	16.5	17.5	17.9	8.9	14.3	18.3	15.2	14.7	16.1	15.4	15.8	11.4
hexyl acetate	1007	1009	0.2	0.5	0.1	0.1	0.4	0.4	0.1	0.1	-	0.2	0.1	0.2	-	0.1	-	0.1
diethyl succinate	1176	1178	1.4	2.7	4.0	2.0	4.3	4.5	2.2	3.2	1.0	2.6	-	1.0	2.6	2.1	2.1	2.1
ethyl octanoate	1196	1204	47.9	48.4	48.0	56.8	47.7	52.7	52.5	49.6	58.1	52.9	55.4	55.6	49.0	50.1	48.6	35.5
isoamyl hexanoate	1252	1250	0.4	-	0.1	-	-	-	0.1	-	0.1	0.1	-	0.1	0.1	0.1	0.1	-
2-phenyl ethyl acetate	1254	1254	0.7	1.0	-	-	0.3	0.2	0.1	-	1.1	-	-	1.2	-	-	0.1	-
ethyl 4*E*-decenoate	1380	1389	-	0.2	0.1	0.1	1.0	0.9	-	4.3	0.4	-	0.2	0.2	0.2	0.2	0.3	0.3
ethyl decanoate	1395	1398	16.5	10.8	0.7	0.3	8.7	8.2	15.3	14.4	13.5	17.6	10.7	14.6	18.4	17.4	18.4	16.1
isoamyl octanoate	1442	1448	0.3	0.1	13.4	8.7	1.1	1.3	0.2	0.1	-	0.2	0.1	-	0.2	0.1	0.3	0.1
ethyl dodecanoate	1594	1582	0.3	0.2	0.4	0.1	-	-	0.8	2.7	-	0.1	0.1	-	0.4	0.2	0.4	0.4
**Monoterpenes**			**2.9**	**3.3**	**3.0**	**1.8**	**0.8**	**0.7**	**0.5**	**3.9**	**4.4**	**0.2**	**4.6**	**4.2**	**2.0**	**0.1**	**0.2**	**1.7**
*cis*-pinane	982	981	-	-	-	-	-	-	-	-	0.4	-	0.3	0.3	-	-	0.1	1.4
terpinolene	1086	1086	0.2	0.1	-	-	-	-	-	-	0.2	-	0.1	0.2	-	-	-	-
linalool	1095	1098	1.6	1.6	0.6	0.3	0.4	0.3	0.2	1.0	0.7	0.2	0.5	1.1	-	-	-	-
hotrienol	1104	1103	0.1	0.1	0.1	0.2	-	-	-	0.4	0.6	-	0.3	0.4	2.0	-	-	0.1
nerol oxide	1154	1154	0.2	0.2	0.1	-	-	-	0.1	0.5	0.7	-	0.6	0.6	-	-	-	-
*cis*-pinane hydrate	1143	1139	0.1	0.2	-	0.1	-	-	-	0.1	0.2	-	0.1	0.2	-	-	-	-
α-terpineol	1186	1200	0.6	0.7	2.0	0.4	0.2	0.1	0.1	0.7	1.2	-	2.2	1.2	-	-	-	0.1
ascaridole	1234	1240	-	-	0.1	0.7	0.3	0.3	-	0.8	0.2	-	0.1	0.3	0.1	-	0.1	0.1
*trans*-geraniol	1249	1246	0.2	0.4	0.1	0.1	-	-	0.1	0.4	0.3	-	0.4	-	-	0.1	-	-
**Alcohols**			**2.5**	**2.8**	**4.6**	**4.3**	**2.7**	**2.6**	**2.4**	**3.0**	**0.8**	**1.5**	**2.8**	**1.7**	**0.4**	**1.7**	**2.0**	**2.2**
*n*-hexanol	863	867	1.1	0.8	1.1	1.3	0.9	1.1	1.1	0.8	0.2	1.0	0.5	0.3	0.3	0.3	0.4	0.6
*n*-heptanol	959	968	0.2	-	-	0.1	-	-	-	0.2	0.5	-	0.4	0.5	-	-	-	0.4
*n*-octanol	1063	1074	0.1	0.1	0.3	0.2	-	-	0.2	0.1	0.1	0.1	0.2	0.1	0.1	0.1	0.1	0.1
2-nonanol	1097	1100	-	-	-	-	-	-	0.1	-	-	-	0.1	0.2	0.1	0.1	-	-
2-phenyl -ethanol	1107	1106	1.1	1.8	3.2	2.7	1.7	1.5	1.2	1.8	-	0.5	1.5	0.6	-	1.2	1.5	1.0
**Ketones**			**0.2**	**-**	**-**	**-**	**-**	**-**	**-**	**0.1**	**0.2**	**-**	**-**	**-**	**0.2**	**0.2**	**0.2**	**5.8**
2-nonanone	1087	1091	0.2	-	-	-	-	-	-	0.1	0.2	-	-	-	**0.2**	0.2	0.2	5.8
**Carboxylic acid**			**-**	**-**	**0.5**	**0.6**	**0.7**	**0.8**	**-**	**-**	**-**	**-**	**-**	**-**	**0.1**	**0.3**	**-**	**-**
hexanoic acid	967	973	-	-	0.5	0.6	0.7	0.8	-	-	-	-	-	-	0.1	0.3	-	-
**Alkenes**			**0.2**	**-**	**-**	**-**	**0.8**	**0.9**	**0.3**	**0.5**	**0.2**	**-**	**0.6**	**0.3**	**0.4**	**0.9**	**0.4**	**0.7**
4*E*-octene	801	800	0.2	-	-	-	0.8	0.9	0.3	0.5	0.2	-	0.6	0.3	0.4	0.9	0.4	0.7

a Linear retention indices.

b Lit.: LRI obtained from literature (Adams, 2017).

c Exp.: LRI experimental obtained by the injection of a homologous series of C_8_–C_20_ *n*-alkanes using the Van den Dool and Kratz equation (Van den Dool and Kratz, 1963). Grape varieties code: MJ = Moscatel de Jundiaí; ME = Moscato Embrapa, M = IAC Madalena; R = IAC-Ribas; 5 = Rootstock IAC 572 Jales; 7 = Rootstock IAC 766 Campinas; J = cultivation site of Jundiaí; V = cultivation site of Votuporanga.

Generally, esters are commonly attributed as the primary aroma of wines, with aromatic notes recognized as apple, grape, banana, wine-like, and fruity, which can even at low concentrations impact the aroma perception of wines.^3,38^ Ethyl octanoate was the most abundant compound in all analyzed wines, ranging from 35.5% to 58.1% of the analyzed aromatic compounds, with the lowest value presented by the wine made with the IAC Ribas grape grafted onto the rootstock IAC 766 and grown in the city of Jundiaí (SJ7). However, this same wine sample showed a higher relative abundance of 2-nonanone (5.8%), which was at least five times higher than that observed for the other samples.

The ester diethyl succinate, characterized by notes of fruity and melon, was present in all samples (except in M5J). Although its concentration has not exceeded 5% of the composition of the wine samples, this compound is associated with its contribution to wine aromas due to its odor threshold of 1.2 mg/L.^39^ Additionally, the compound is derived from malolactic fermentation, a process conducted by malolactic bacteria, which is used to control the acidity of wine. This process results from the decarboxylation of l-malic acid into l-lactic acid.^40^

Some substances were restricted to a few samples, such as the compound terpinolene, a monocyclic monoterpene containing a cyclohexene ring, characterized by its distinctive pine fragrance.^41^ This compound was present in the wines M5J, M7J, MJ7V, and MJ5V, which were produced from different rootstocks, suggesting that this volatile compound may have resulted from the metabolism of the specific grape varieties used in the wine production (IAC Madalena and Moscatel de Jundiaí). The compound isoamyl acetate, however, observed in almost all analyzed wines, is responsible for 12% of the MJ7V wine aroma, whereas it was not detected in MJ7J and only 0.8% was observed in M5V wine. Such variance is relevant to afford wine with different sensory features, as this substance is known to produce an olfactory experience similar to banana aromas, a desirable characteristic due to its contribution of the sweetest aroma commonly sought in this type of wine.

In addition, monoterpenes as linalool, terpinolene, α-terpineol, *cis*-pinane, nerol oxide, ascaridol, hotrienol, and *trans*-geraniol were observed in the samples in substantial amounts, as disclosed in Table 2.^42^ The presence of these compounds in white wines is pivotal even in low amounts, as these are the main compounds responsible for the essential characteristics in wines made through the fermentation of white grapes, such as soft and sweet aromatic notes, which refer to the aromas of flowers and fruits, with a great capacity for sensory stimulation.

### 3.3 Principal Component Analyses (PCA) of Wine Aromas

Principal component analysis (PCA) was applied to the volatile organic compounds (VOCs) of 16 wines produced from combinations of scion, rootstock, and cultivation site. The first two components account for 38.7% (Figure 2).

**Figure 2:**
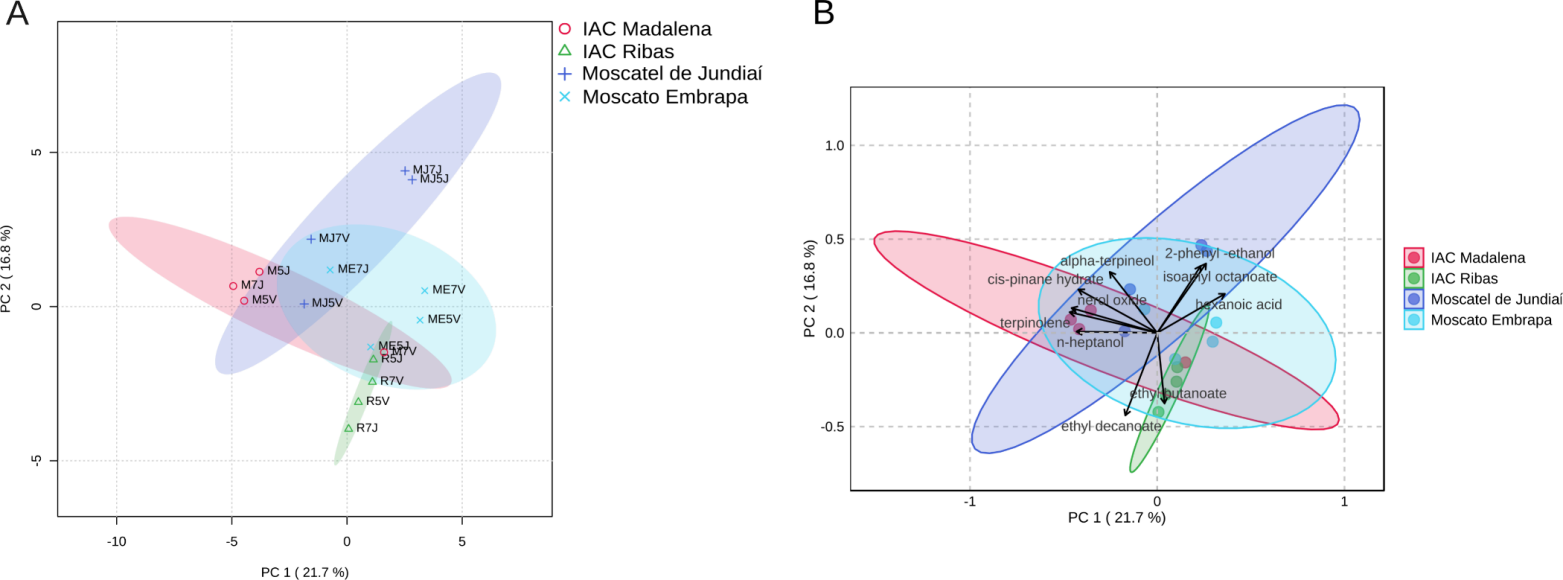
Principal component analysis (PCA) of volatile organic compounds (VOCs) of 16 wines produced from combinations of scion, rootstock, and cultivation site. A) Score plot. B) Biplot.

Multivariate analysis revealed that genetic factors had a more significant influence on the observed variation in volatile organic compounds (VOCs) than environmental factors. The samples clustered primarily according to the four cultivars studied (Figure 2A), demonstrating a lesser influence of the growing environment (city) and rootstock on volatile composition. Among the cultivars, IAC Ribas (R) showed the most stability, with the least variation in VOC levels among its samples. PERMANOVA confirmed that variety was the only significant factor affecting volatile composition (*p* = 0.027), while rootstock (*p* = 0.998) and location (*p* = 0.381) showed no significant effects (Table 3).

**Table 3:** PERMANOVA Results for Volatile and Phenolic Compounds of White Wines Made from the Combination of Different Vine Varieties, Rootstocks, and Cultivation Sites, in São Paulo State, Brazil

compounds	factor	*p*-value
VOCs	variety	0.027^a^
	rootstock	0.998
	location	0.31
Phenolics	variety	0.002^b^
	rootstock	0.839
	location	0.291

a *p* < 0.05.

b *p* < 0.01.

In general, although genetics was the primary factor responsible for sample separation, a secondary influence of cultivation location was observed in the cultivar Moscatel de Jundiaí (MJ) grafted onto the rootstocks IAC 572 and IAC 766 (MJ5J and MJ7J). The samples grown in Jundiaí showed greater similarity, a behavior also observed in samples of this cultivar obtained in Votuporanga. A similar trend was observed for Moscato Embrapa (ME). Among the sources of variation (cultivar × location × rootstock), the rootstock demonstrated the least impact on the chemical composition of volatile compounds. The results obtained are in agreement with the fact that genetic variability among grape cultivars directly influences flavor and color profiles, which are linked to polyphenol content.^43^ Additionally, the cultivation environment and choice of rootstocks significantly impact grape quality by controlling the accumulation of bioactive compounds, including phenolics, anthocyanins, and volatile compounds. Studies have shown that rootstock and training systems impact physicochemical parameters, including pH and acidity, which are closely related to grape quality^43^.

The vectors of the substances that contributed most to the separation between the groups are presented in the biplot (Figure 2B). The esters ethyl butanoate and ethyl decanoate showed a strong association with the IAC Ribas cultivar. At the same time, 2-phenyl ethanol, isoamyl octanoate, and hexanoic acid were more closely related to the Moscatel de Jundiaí cultivar. Compounds such as terpinolene, n-heptanol, myrtenol oxide, and α-terpineol were predominant in samples of the IAC Madalena cultivar.

The heatmap presented in Figure 3A,B confirm and complement the PCA analysis, demonstrating that the observed variations are primarily due to the differences between the canopy cultivars. Moscato Embrapa stands out for its volatile profile with a strong association with esters and ketones, such as hexyl acetate, n-hexanol, hexanoic acid, diethyl succinate, ehtyl dodecanoate, ethyl 4 (*E*)-decanoate and ethyl butanoate, which confer markedly fruity and sweet characteristics,^43,44^ and can be desired in wines with a tropical aromatic appeal.

**Figure 3:**
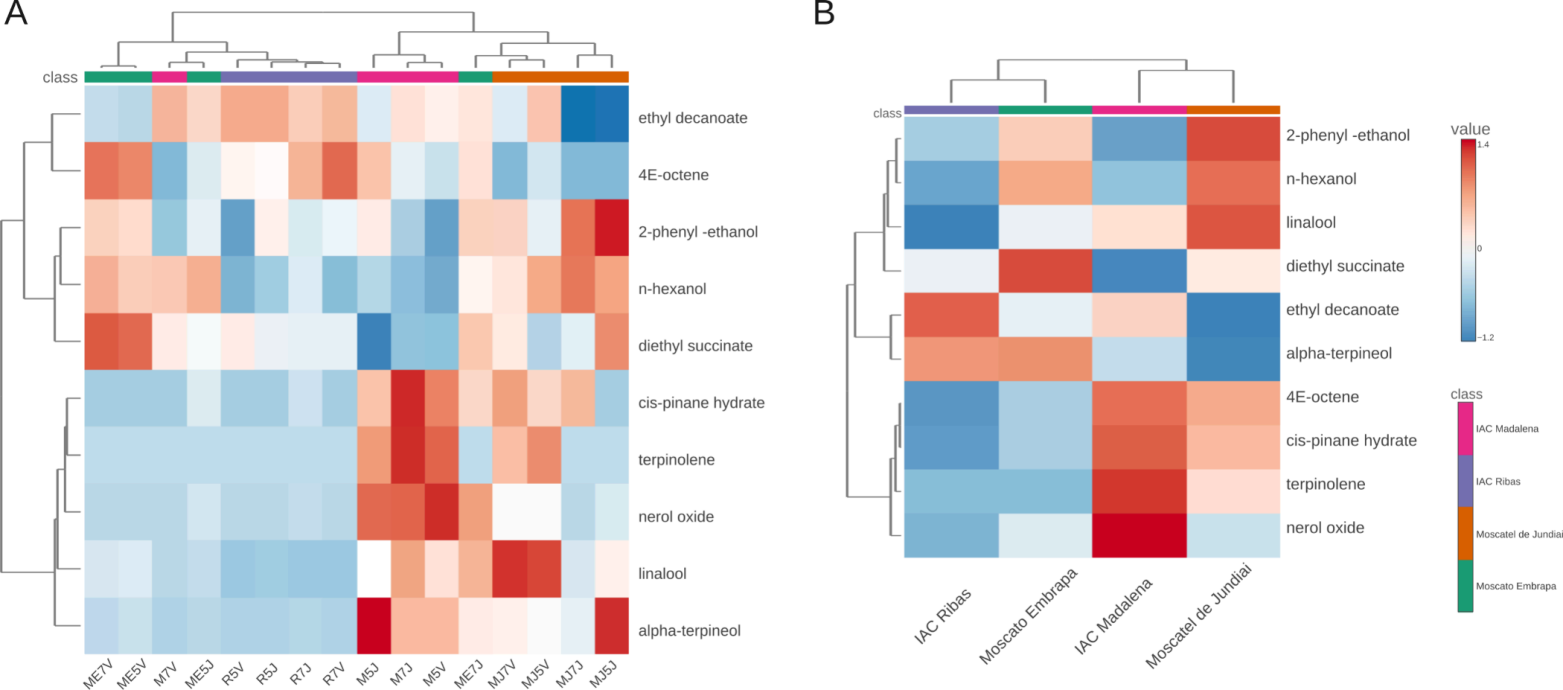
Dendrogram and heat map analysis by sample and by cultivar of the volatile compounds identified and quantified in the wines. A) Concentration in each sample. B) Concentration summarized by the mean values for each wine variety.

### 3.4 High-Performance Liquid Chromatography with Diode Array Detection (HPLC-DAD) Analyses of Phenolic Compounds

The phenolic composition of the wines was analyzed by HPLC-DAD, and the results are presented in Table 4, organized by chemical class (phenolic acids, stilbenes, flavanols, and flavonols).

**Table 4:** Phenolic Chemical Composition of White Wines Made from the Combination of Different Vine Varieties, Rootstocks, and Cultivation Sites, in São Paulo State, Brazil^a^

	samples (mg/L)															
compounds	MJ5V	MJ7V	MJ5J	MJ7J	ME5V	ME7V	ME5J	ME7J	M5V	M7V	M5J	M7J	R5V	R7V	R5J	R7J
**Phenolic Acids**																
Gallic acid	5.17	-	4.08	4.05	-	-	-	-	-	-	-	-	-	-	-	-
Syringic acid	-	2.01	0.43	0.05	-	-	0.03	0.04	0.09	0.11	-	0.05	0.07	0.08	0.09	0.07
Caftaric acid	25.17	31.11	18.62	12.29	149.48	175.73	64.21	43.66	39.81	32.98	54.77	43.75	35.53	32.97	31.59	22.29
Chlorogenic acid	1.29	1.97	0.64	0.38	5.87	5.26	3.11	2.36	2.78	2.43	4.20	4.55	4.21	3.51	5.09	2.91
Cafeic acid	0.60	0.58	0.92	1.57	1.08	2.06	2.01	1.54	0.72	0.89	0.47	0.30	0.47	0.69	0.56	0.79
*p*-Coumaric acid	0.02	0.04	-	0.02	-	0.09	0.15	0.10	0.15	0.21	0.02	-	0.15	0.33	0.25	0.26
**Stilbenes**																
*trans*-Resveratrol	-	-	-	-	-	-	-	-	-	-	-	-	-	-	-	-
*cis*-Resveratrol	-	-	-	-	-	-	-	-	0.28	0.28	0.41	0.35	0.34	0.39	0.61	0.57
**Flavanols**																
Procyanidin B1	0.87	1.63	0.66	0.87	0.54	0.84	0.66	0.61	0.60	0.82	0.76	0.39	0.62	0.70	0.85	0.58
Catechin	4.90	7.98	2.79	1.29	0.75	0.76	0.53	0.57	1.20	1.36	1.85	0.82	1.41	1.38	1.17	0.88
Procyanidin B2	1.19	2.27	0.82	0.68	0.45	0.82	0.91	0.79	1.32	1.43	1.61	1.79	1.43	1.47	2.47	2.15
Epig. Gallate^a^	0.56	0.75	0.81	1.35	0.89	1.75	1.60	1.21	0.61	0.69	0.43	0.28	0.39	0.55	0.38	0.60
Epicatechin	-	4.76	1.11	-	0.09	-	0.15	0.15	0.19	-	0.47	-	-	-	-	0.13
Epic. gallate^b^	-	-	0.29	-	0.25	0.37	0.48	0.39	0.25	0.29	-	-	-	0.33	0.28	0.28
Procyanidin A2	2.1	1.75	1.51	0.61	3.23	4.01	5.00	6.31	1.78	1.61	1.44	2.09	1.68	1.01	0.94	1.02
**Flavonols**																
Qc 3-glc^c^	-	0.15	-	-	-	-	-	-	-	-	-	-	0.14	0.16	0.12	-
Rutin	-	-	-	-	-	-	-	-	-	-	-	-	-	-	-	-
Kaempferol	0.02	-	-	-	-	-	-	-	-	-	-	-	-	-	-	-

a Grape varieties code: MJ = Moscatel de Jundiaí; ME = Moscato Embrapa, M = IAC Madalena; R = IAC-Ribas; 5 = Rootstock IAC_572 Jales; 7 = Rootstock IAC_766 Campinas; J = cultivation site of Jundiaí; V = cultivation site of Votuporanga. a) Epig. Gallate: Epigallocatechin gallate; b) Epic. gallate: Epicatechin gallate; c) Qc 3-glc: Quercetin 3-glucoside.

From these analyses, phenolic acids represented the class of metabolites present at the highest concentration in the samples, with the highest concentration observed in ME7V at up to 175.7 mg/L. These results are in agreement, as white grapes synthesize a lower amount of flavanols compared to red grapes, leading to a lower concentration of these compounds and, consequently, a different color of the berries.^38,45^

Thus, the grape variety that showed the highest concentration of these compounds was Moscato Embrapa, particularly those grown in the city of Votuporanga (ME5V and ME7V), due to the highest concentration of caftaric acid (Table 4). Moreover, another relevant aspect of the wine’s phenolic composition, as observed through HPLC-DAD analyses, was the presence of the stilbene *cis*-resveratrol in the IAC Madalena and IAC Ribas grape cultivars. This compound is considered desirable and an essential constituent of wines due to its cardioprotective effects,^46^ providing a medicinal value to wines made from these varieties.

Although *trans*-resveratrol was not detected in any of the analyzed white wine samples, its absence is consistent with previous reports indicating that this isomer is significantly more prevalent in red wines due to extended skin maceration during vinification and its high concentration in grape skins compared with white wines.^47^ In white winemaking, limited skin contact, oxidative conditions, and isomerization processes may promote degradation or conversion of *trans*-resveratrol into its *cis* form, which has been documented to occur under environmental stress and specific conditions influencing stilbene equilibria.^48^

### 3.5 Principal Component Analysis (PCA) of Phenolic Chemical Composition of Wines

Principal Component Analysis (PCA) was conducted to investigate the phenolic composition of wines produced from four grape cultivars grafted onto two different rootstocks and cultivated in the regions of Votuporanga and Jundiaí. The two-component PCA model explained 56.7% of the total variance, with the first principal component (PC1) accounting for 30.8% and the second (PC2) for 25.9% of the variance (Figure 4A,B) . This multivariate approach enabled the identification of compositional patterns among the wine samples, revealing clustering primarily based on grape cultivar, independent of cultivation site or rootstock, and highlighting the predominant influence of genetic factors. PERMANOVA demonstrated a highly significant variety effect (*p* = 0.002), explaining 59% of the variance. Rootstock (*p* = 0.839) and location (*p* = 0.291) were not significant (Table 3).

**Figure 4:**
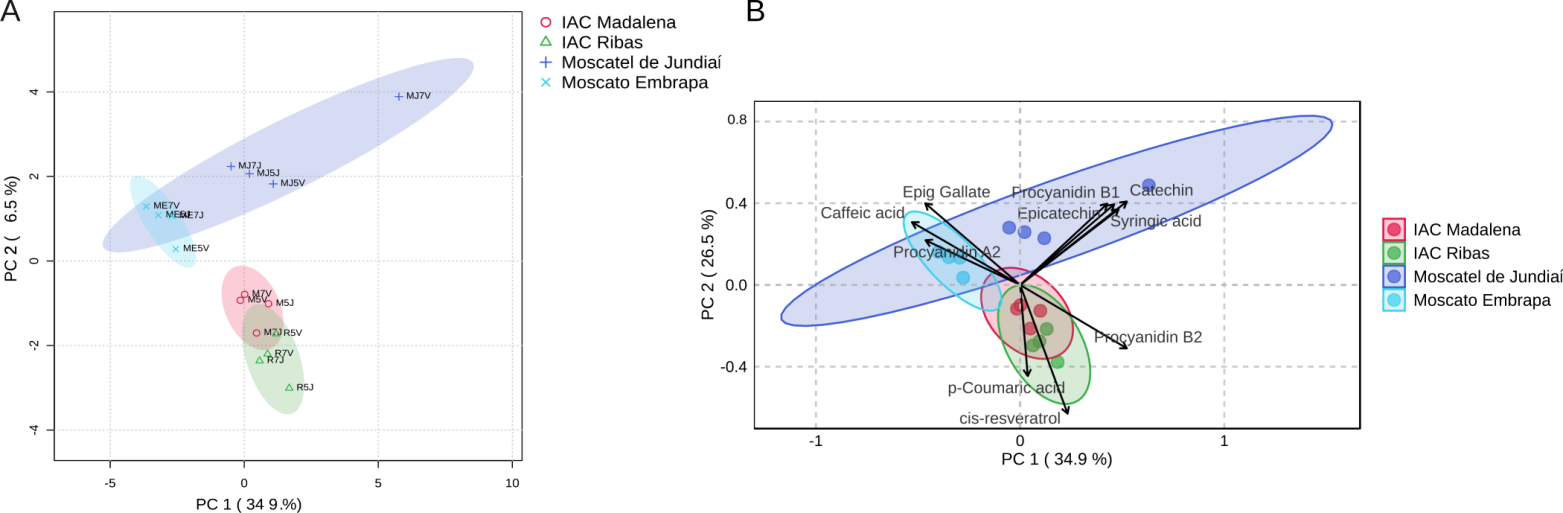
Principal component analysis (PCA) of phenolic chemical composition of 16 wines produced from combinations of scion, rootstock, and cultivation site.

PC1 was positively correlated with quercetin-3-glucoside, catechin, procyanidin B1, syringic acid, and epicatechin, while showing negative correlations with caffeic acid, epigallocatechin gallate, epicatechin gallate, and procyanidin A2. Meanwhile, PC2 showed positive correlations with *cis*-resveratrol, *p*-coumaric acid, and chlorogenic acid, and negative correlations with gallic acid, catechin, and epigallocatechin gallate.

The Principal Component Analysis (PCA) (Figure 4A) revealed the formation of two main groups. The first group comprises the cultivars Moscato Embrapa and Moscatel de Jundiaí, grafted onto various rootstocks and cultivated in distinct locations. It was observed that the Moscatel de Jundiaí cultivar grafted onto IAC 766 and grown in Votuporanga stood out as the most divergent among the others. When analyzing the biplot (Figure 4B), this cultivar showed the highest concentrations of catechin, procyanidin B1, epicatechin, and syringic acid. The combinations of the ME and MJ cultivars grafted onto rootstocks 766 and 572, cultivated in Jundiaí and Votuporanga, exhibited higher concentrations of epigallocatechin, caffeic acid, and procyanidin A2.

On the other hand, the combinations formed by the cultivars IAC Ribas and IAC Madalena, associated with their respective rootstocks and cultivation sites, presented higher levels of procyanidin B2, *p*-coumaric acid, and cis-resveratrol. Although these are white grape cultivars intended for wine production, they showed significant levels of cis-resveratrol. The phenolic profiles exhibited limited variation, attributable to neither cultivation site nor rootstock. The most notable intravarietal divergence was observed in the Moscatel de Jundiaí sample grown in Votuporanga on rootstock 766. This sample differed primarily due to the absence of gallic acid and higher concentrations of caftaric and chlorogenic acids, suggesting that, while minor, specific rootstock-environment interactions can influence certain phenolic markers.

The IAC Ribas and IAC Madalena cultivars exhibited greater chemical similarity, with *cis*-resveratrol identified exclusively in both, suggesting shared biosynthetic traits or similar genetic expression profiles related to stilbene synthesis. Conversely, the compounds epicatechin gallate and procyanidin A2 were distinctive of the Moscato Embrapa samples, effectively differentiating them from the other cultivars (Figure 5).

**Figure 5:**
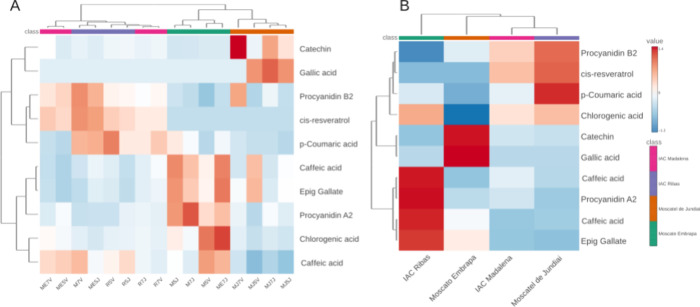
(A, B) Dendrogram and heat map analysis by sample and by cultivar of the phenolic chemical composition of 16 wines produced from combinations of scion, rootstock, and cultivation site.

Overall, the data suggest that the phenolic composition of wines derived from white grape cultivars is primarily governed by genetic factors, with comparatively low phenotypic plasticity in response to environmental conditions or the rootstock used. This behavior was also observed for volatile compounds, whose composition is generally more responsive to edaphic and climatic conditions. Therefore, genetic factors emerge as the primary determinant of the phenolic composition in the evaluated samples.

## 4 Conclusion

This study analyzed the metabolic profiles of white wines, focusing on their total phenolic content, volatile organic compounds (VOCs), and phenolic chemical composition. Wines from the Moscato Embrapa (ME) and Moscatel de Jundiaí (MJ) cultivars exhibited the highest levels of total phenolic content. In contrast, wines from the IACMadalenaand IACRibas cultivars showed a more stable phenolic composition, with minimal variation across different cultivation sites and rootstocks.

The analytical experiments and statistical analyses, supported by both PCA and PERMANOVA, demonstrated that variations in wine aromas and phenolic profiles were primarily influenced by the grape cultivars used. However, the site of cultivation also had a significant impact on the aroma profiles of certain wines, highlighting the role of environmental factors in shaping sensory characteristics.

Ethyl esters were the dominant class of aromatic compounds across all wine samples, with ethyl octanoate being the most abundant, ranging from 35.5% to 58.1%. Additionally, nine monoterpenes were identified in the aroma composition. Given their low sensory thresholds, these compounds likely contribute floral and fruity notes, enhancing the overall aromatic complexity of the wines.^49,50^

HPLC-DAD analysis revealed that flavanols and phenolic acids were the major constituents in the phenolic composition. Caftaric acid was the most prevalent compound in all wine samples, particularly in wines from theMoscato Embrapa cultivar, which contained concentrations up to 175.73 mg/L. This cultivar also exhibited higher levels of chlorogenic acid, caffeic acid, and procyanidin A2, distinguishing it for its ability to produce these specific phenolic compounds. IAC Ribas and IAC Madalena cultivars were notable for their exclusive presence of cis-resveratrol, a relevant marker from both functional and nutritional perspectives. The compositional stability of the latter also highlights their suitability for winemaking under different growing conditions.

These findings provide valuable insights into the quality determinants of wines produced in São Paulo state, offering new possibilities for grape cultivation in tropical climates, such as those found in southeastern Brazil. Moreover, this study lays the groundwork for the development of stable, competitive wine products that can perform well in global markets, including the emerging Brazilian wine industry, which remains predominantly concentrated in the southern region of the country.
